# De Novo Valve Tissue Morphology Following Bioscaffold Mitral Valve Replacement in a Juvenile Non-Human Primate Model

**DOI:** 10.3390/bioengineering8070100

**Published:** 2021-07-16

**Authors:** Brittany A. Gonzalez, Marcos Perez Gonzalez, Frank Scholl, Steven Bibevski, Elena Ladich, Jennifer Bibevski, Pablo Morales, Jesus Lopez, Mike Casares, Vincent Brehier, Lazaro Hernandez, Sharan Ramaswamy

**Affiliations:** 1 Department of Biomedical Engineering, Florida International University, Miami, FL 33174, USA; bgonz049@fiu.edu (B.A.G.); mgonz862@fiu.edu (M.P.G.); SBibevski@mhs.net (S.B.); 2 Memorial Healthcare System, Joe DiMaggio Children’s Hospital, Hollywood, FL 33021, USA; fScholl@mhs.net (F.S.); elenarladich@gmail.com (E.L.); mcasares@ccsperfusion.com (M.C.); brehierv@yahoo.com (V.B.); LazHernandez@mhs.net (L.H.); 3 COR Veterinary Surgery Services, Hollywood, FL 33301, USA; jennifer.bibevski@yahoo.com; 4 The Mannheimer Foundation, Inc., Homestead, FL 33034, USA; pmorales@mannheimerfoundation.org (P.M.); jlopez@mannheimerfoundation.org (J.L.)

**Keywords:** porcine small intestinal submucosa (PSIS), non-human primate model, mitral valve, de novo valve tissues, spatial intensity mapping, extracellular matrix, unfilled tissues

## Abstract

The utility of implanting a bioscaffold mitral valve consisting of porcine small intestinal submucosa (PSIS) in a juvenile baboon model (12 to 14 months old at the time of implant; *n* = 3) to assess their in vivo tissue remodeling responses was investigated. Our findings demonstrated that the PSIS mitral valve exhibited the robust presence of de novo extracellular matrix (ECM) at all explantation time points (at 3-, 11-, and 20-months). Apart from a significantly lower level of proteoglycans in the implanted valve’s annulus region (*p* < 0.05) at 3 months compared to the 11- and 20-month explants, there were no other significant differences (*p* > 0.05) found between any of the other principal valve ECM components (collagen and elastin) at the leaflet, annulus, or chordae tendinea locations, across these time points. In particular, neochordae tissue had formed, which seamlessly integrated with the native papillary muscles. However, additional processing will be required to trigger accelerated, uniform and complete valve ECM formation in the recipient. Regardless of the specific processing done to the bioscaffold valve, in this proof-of-concept study, we estimate that a 3-month window following bioscaffold valve replacement is the timeline in which complete regeneration of the valve and integration with the host needs to occur.

## 1. Introduction

Heart valve disease remains a major healthcare concern in the cardiovascular field. Adult patients now potentially have minimally invasive transcatheter treatment options in addition to open-heart surgery, particularly the area of aortic valve replacement. On the other hand, minimally invasive approaches are utilized less for treatment of a critically diseased mitral valve. Primarily, this is owing to the use of mitral valve repair for treatment in a majority of cases, with a very high rate of success post-surgery [1]. However, exceptions to these are cases of severe mitral valve annular calcification and regurgitation, wherein surgical complications such as paravalvular leakage arise and several subsets of these patients suffer from comorbidities, thereby making surgery a risky option [1]. While the advancement therefore with minimally invasive approaches for the treatment of adult mitral valve diseases is actively being pursued, the treatment of critical mitral valve defects in the pediatric population remains a serious challenge with no acceptable treatment that is currently available.

Congenital heart valve defects (CHVDs) are diagnosed as a problem with the structure or function of the heart valve at birth or soon after, which leads to abnormal blood flow. Congenital heart malformations are the most common type of defect present at birth, with CHVDs occurring in roughly 2% of live births, although the incidence is thought to be significantly higher since many cases remain subclinical and are unidentified [2]. There are several types of CHVDs, ranging from simple to complex and critical. Infants born with critical congenital heart valve defects (CCHVDs) have no viable treatment and suffer from a high rate of mortality and with surgeries required within the first year of their birth (www.nhlbi.nih.gov). In the case of the critical mitral valve defects in children, as with adults, repair is always the preferred option, when possible, over valve replacement. However, CCHVDs of the mitral valve in pediatric patients necessitate valve replacement in conditions such as rheumatic disease, endocarditis, Shone syndrome-related mitral stenosis, or when the repair fails [3]. Given the small sizing of the patients at birth, and complications that arise with an oversized valve prosthesis (e.g., sub-aortic obstruction [3]), mitral valve replacement remains a very limited treatment option, unless a heart transplant is available. For this reason, mitral valve replacement in children suffering from CCHVDs has a high mortality rate, with approximately 67% of patients not surviving beyond 5 years after valve replacement surgery [3].

Efforts to replace a patient’s defective mitral valve with their own pulmonary valve, i.e., Ross II procedure in young adults, have exhibited very promising results in terms of long-term valve function after securement in the systemic circulation [4]. However, Ross-based surgeries unfortunately have a much higher risk of homograft failure when the patient is younger and/or smaller in size [4]. Nonetheless, evidence of homograft, i.e., pulmonary heart valve tissue adaptation to the systemic circulation suggests that valves have the capacity to remodel their extracellular matrix (ECM) to facilitate adequate function [5]. Therefore, a tissue-engineered heart valve (TEHV) can potentially be an ideal treatment option for young pediatric patients suffering from CCHVDs, where somatic growth support and biological integration of the implanted heart valve are possible. This could eliminate the need for multiple reoperations as the patient grows and matures, and at minimum, may serve as a bridge-to-prosthetic-valve replacement when sufficient patient growth has occurred. Specifically, the area of greatest need for a functional TEHVs could revolutionize treatment, and alter the current trend of a poor prognosis, would be in infants born with critical mitral valve defects, when mitral valve repair is not feasible. 

For TEHVs, bioscaffolds have been recently investigated owing to their viscoelastic mechanical deformation responses similar to the healthy valve ECM. These bioscaffolds are typically decellularized, i.e., the tissue or organ is removed of its cellular components, while retaining the ECM structure and its proteins [6]. Similar to bioprosthetic valves, decellularized heart valves provide hemodynamics mimicking the native heart valves. Furthermore, decellularized heart valves have the added advantage of not being treated with glutaraldehyde, which allows for the host’s native cells to repopulate and remodel [6] the implanted construct. A decellularized material that has recently received considerable attention for use in cardiovascular applications, and which has also received Food and Drug Administration (FDA) Investigational Device Exemption (IDE) for early clinical feasibility assessment, is porcine small intestinal submucosa (PSIS) [7,8,9,10,11,12,13,14,15,16,17,18,19,20,21,22,23,24,25,26,27,28,29,30,31,32].

Of note, preclinical and clinical cardiovascular studies have reported both positive [16,18,26,33,34] and negative [14,20,21,25,27,28,35,36,37,38] outcomes with PSIS. Some of the major concerns of PSIS are associated with an adverse immune response (e.g., chronic inflammation) or acute functional failure associated with construct integrity, such as tissue shrinkage [21,25,27,28,36,37]. In contrast, early in vivo heart valve studies in the ovine model using PSIS resulted in promising matrix repopulation with native cells, including surface endothelialization. On the other hand, a recent study on PSIS pulmonary valve replacement in sheep and in lambs exhibited xenogeneic hostile immune responses by way of inflammation and endocarditis that led to premature PSIS pulmonary valve failure [39]. However, it has been observed that ovine models are unable to consistently mimic the human responses for heart valves, as their transvalvular gradients are lower than that of humans [14,20,27,35]. A non-human primate model, if available, would therefore be useful is assessing a more realistic response to PSIS valves. Currently human clinical trials with PSIS usage for the valve application have been promising, with only trivial and mild regurgitation observed in patients who underwent PSIS valve repair [18,27]. In addition, even though it is relatively recent, there is currently an ongoing clinical trial on PSIS valve replacement in adults suffering from a dysfunctional tricuspid heart valve (Cormatrix, Cor TRICUSPID ECM Valve, Roswell, GA, USA; clinicaltrials.gov). 

The application of the current study is in the area of regenerative treatment for critical congenital heart valve defects in the children, specifically in the mitral position. We previously showed that PSIS mitral valves have proven to function in a juvenile (12–14-month-old) baboon model in the acute timeframe [30,31,32]. However, it was found that the altered, post-fatigue, structural responses of PSIS, rather than de novo tissue formation, were primarily responsible for the valve’s ability to accommodate growth following PSIS mitral valve replacement. Therefore, the goals of the present study were to (i) identify the nature of de novo valve tissues that were produced in vivo following the implantation of raw PSIS bioscaffold mitral valves in a juvenile, non-human primate model, via histological and immunohistochemical characterization of the new valve ECM that had formed, and (ii) to quantify a time-window in which complete host valve regeneration would be needed when bioscaffold valve replacements are used [40].

## 2. Materials and Methods

### 2.1. In Vivo Pilot Assessment of PSIS Mitral Valve 

As we previously had reported [30,31,32], in a pilot assessment of PSIS valve function and somatic growth, three male juvenile (12 to 14 months old) hamadryas baboons (Papio hamadryas) underwent mitral valve replacement surgery. All procedures were performed in accordance with the Institutional Animal Care and Use Committees from Florida International University (IACUC-16-036-CR02) and the Mannheimer Foundation, Inc. (IACUC no. 2015-07). All breeding, housing, and in vivo procedures were performed at the Mannheimer Foundation, Inc. (Homestead, FL, USA). The in vivo procedures were performed by pediatric cardiac surgeons (Joe DiMaggio’s Children Hospital, Hollywood, FL, USA).

### 2.2. Surgical Preparation and Procedure for PSIS Mitral Valve Implantations

**Figure 1:**
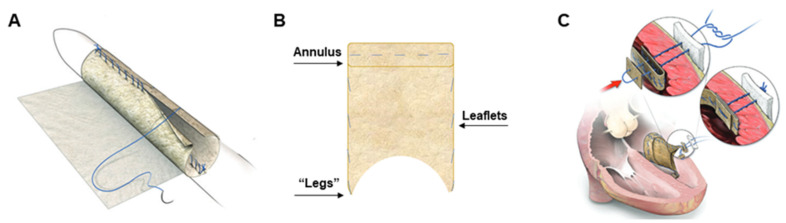
Porcine Small Intestinal Submucosa (PSIS) hand-made mitral valve schematic and implantation points. The PSIS sheets are made into mitral valves (**A**) consisting of three main areas of (**B**) interest, the leaflets, annulus and the “legs”. Once implanted (**C**), the leaflets are the components that open and close as the heart is being pumped. The annulus is a thicker area of the valve that allows the opening for blood to flow through and divides the left atrium and left ventricle. The distal limbs or “legs” of the PSIS mitral valves are attached to the papillary muscles and heart wall when implanted (**C**), functioning similar to the chordae tendineae. The blue lines on the hand-made PSIS mitral valve signify the sutures (**A**,**C**). Panels (**A**,**C**) are reused by permission of Oxford University Press [29].

The surgical preparation and procedures were reported previously [32,40]. In brief, the juvenile baboons were sedated, intubated, placed on a respiratory ventilator, and subsequently given antibiotics an hour prior to the incision time. General endotracheal anesthesia was administered, prepared, and draped with normal sterile techniques (chlorhexidine-based solution). Immediately before implantation, echocardiography was used to measure the native heart valve annulus and leaflet length to manually reconstruct a custom-made bi-leaflet mitral valve using PSIS (2-ply PSIS sheet, Cormatrix, Roswell, GA, USA), for each of the three animal subjects [24,29]. Once the baboons were prepared and draped, a right thoracotomy was performed and the animals were heparinized, placed on cardiopulmonary bypass and cooled to 34 °C (mild hypothermia). Thereafter, vents, needles, caval snares, and a cross-clamp were placed. The interatrial groove was dissected, and the left atrium was accessed through the left ventricular free wall. Next, the native mitral valve was excised, including the chordal attachments, while the handmade PSIS valve was implanted [29,32]. The PSIS valve apparatus (Figure 1) was attached from its distal limbs or “legs” component, to the papillary muscles as well as to the immediately adjacent left ventricular free wall; thereafter, the orifice of the valve was sewn into the native annulus [29,32]. The heart was then de-aired via an antegrade cardioplegia needle, and the cross-clamp was removed. The incisions were sutured to close surgical access points, and the animals were weaned from cardiopulmonary bypass for recovery. Anticoagulants (aspirin, 325 mg daily) were given to the baboons orally for 30 days following PSIS valve implantation.

### 2.3. Histological and Immunostaining of Explanted PSIS Mitral Valves for Assessment of Somatic Growth and Extracellular Content

**Figure 2:**
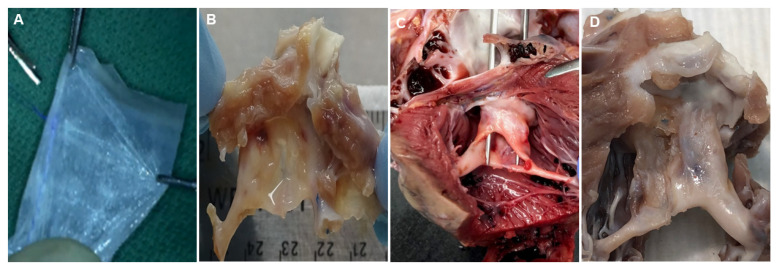
Porcine Small Intestinal Submucosa (PSIS) hand-made mitral valve explants. PSIS mitral valves (**A**) before implantation and (**B**) 3 months, (**C**) 11 months, and (**D**) 20 months post-implantation. There was (**B**–**D**) new tissue formation clearly observed on the explanted PSIS valves compared to the (**A**) original PSIS bioscaffold before implantation.

PSIS mitral valves were explanted from the juvenile baboons (*n* = 3) at 3-, 11- and 20-months post-implantation (Figure 2). The timing of these explants was not intentional but was a result of unexpected and sudden acute, critical PSIS mitral valve insufficiency or failure, necessitating euthanasia of the animals. The specific reasons for PSIS mitral valve failures were due to endocarditis (3-, 20-month explant; Figure 2B,D) that has been previously observed in PSIS pulmonary valve replacement in both adult and juvenile ovine models [39] as well as fibrotic scar tissue adhesions that caused a hole in the posterior leaflet (11-month explant; Figure 2C).

The PSIS valves were subsequently fixed in 10% w/v formalin for 24 h immediately after explanation. The valves were then rinsed with PBS and were subjected to in-depth histological processing (Alizee Pathology LLC., Thurmont, MD, USA). Specifically, Hematoxylin and Eosin (H&E; Alizee Pathology LLC., Thurmont, MD, USA) and Movat’s Pentachrome (Movat’s; Alizee Pathology LLC., Thurmont, MD, USA) stains were performed on native juvenile baboon mitral valves (14-month old; positive control), explanted PSIS mitral valves (3-, 11- and 20-month post-implantation) and unimplanted, decellularized raw, PSIS bioscaffold (CorMatrix, Roswell, GA, USA; negative control) to visualize the cellular content, morphology and extracellular matrix of the tissues. 

Furthermore, immunohistochemistry was completed to assess valve specific cells, including interstitial (smooth muscle actin, α-SMA) and endothelial (cluster of differentiation 31, CD31) cells as well as troponin (Alizee Pathology LLC., Thurmont, MD, USA) to assess the integration of the implanted PSIS valve with the heart [40]. The focal points of the explanted PSIS mitral valves included the leaflets, annulus, and connections to the papillary muscles, which were independently verified by a pathologist in our group (Memorial Healthcare System, Hollywood, FL, USA). All histological and immunohistochemistry images were captured with bright-field microscopy (Zeiss, Axiovert 40 CFL, Maple Grove, MN, USA; Olympus, BX43, Center Valley, PA, USA).

### 2.4. Spatial Intensity Mapping of Explanted PSIS Mitral Valves for Assessment of Percent Extracellular Matrix Content

A Movat’s histological image of the native mitral valve of a juvenile baboon (14 months old) was used as a reference image for all subsequent Movat’s images. The reference image was thresholded for brightness and contrast automatically (ImageJ, NIH Image, Bethesda, MD, USA; accessed on January 2020–July 2021). All the images were then normalized to the thresholded reference image through an in-house script (MATLAB, MathWorks, Inc., Natick, MA, USA). After normalizing, a color segmentation plugin was used (ImageJ, NIH Image, Bethesda, MD, USA; accessed on January 2020–July 2021) and different colors of yellowish-green, purple-black, bluish-green, and fuchsia were chosen to distinguish the different ECM components of collagen, elastin, proteoglycans, and fibrin, respectively. Once completed, the option for independent color channels was selected, and a Hidden Markov Model was used to quantify the resulting percentages of the ECM concentrations. The images were then processed for their respective signal intensities within each ECM component of interest (MATLAB, The MathWorks, Inc., Natick, MA, USA; accessed on January 2020–July 2021) and spatial intensity maps for each of these ECM components were subsequently generated [40]. The average percent (%) ECM component ± the standard error of the mean (SEM) was computed and compared. 

The percent (%) of unfilled PSIS was calculated by first determining the total area of interest in the image (leaflet, annulus, neochordae; ImageJ, NIH Image, Bethesda, MD, USA; accessed on January 2020–July 2021). Next, the total area of remaining PSIS, which was exhibited by a dark pink stain, from each H&E image was calculated (ImageJ, NIH Image, Bethesda, MD, USA). Then, the total area of the remaining PSIS was divided by the total area of interest to compute the average percent (%) of unfilled tissue ± the standard error of the mean (SEM) [40].

### 2.5. Statistical Analysis

The spatial intensity maps were compared based on the location of the tissue and reported for each of the major valve ECM components, i.e., collagen, elastin, proteoglycans, and fibrin, as a function of the total ECM content. The percentage of unfilled de novo tissue (residual PSIS) was computed by determining the area of unfilled tissue as a function of the total ECM content (ImageJ, NIH Image, Bethesda, MD, USA; accessed on January 2020–July 2021). ECM concentrations and unfilled de novo tissue percentages were statistically analyzed using a one-way analysis of variance (ANOVA), with a Dunnett’s test (Minitab, Inc., State College, PA, USA; accessed on January 2020–July 2021) for the leaflet comparisons, a one-way analysis of variance (ANOVA), with a Tukey’s post hoc test (Minitab, Inc., State College, PA, USA; accessed on January 2020–July 2021) for the annulus comparisons and a two-sample *t*-test (Minitab, Inc., State College, PA, USA; accessed on January 2020–July 2021) for valve connections to the papillary muscle comparisons. Statistical significance between any two given groups was found when the *p*-value was less than 0.05 (*p* < 0.05).

## 3. Results

### 3.1. Microstructure and Phenotype of De Novo Valve Tissues on Explanted PSIS Mitral Valves

#### 3.1.1. Cellular Infiltration and Morphology of Explanted Native and PSIS Mitral Valve Leaflets

**Figure 3:**
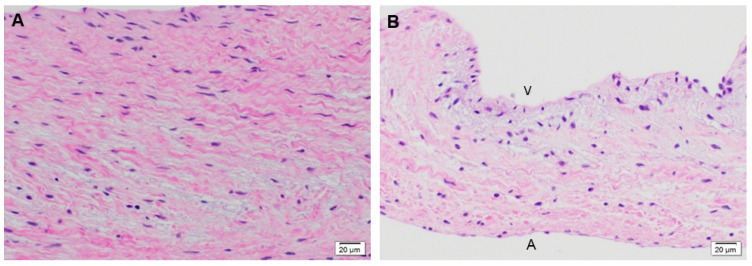
Native juvenile baboon mitral valve explant at 14-months stained with H&E. Histological image of native mitral valve leaflets (**A**,**B**) at 40× demonstrating the normal presence of cells on the leaflet. The label, “A” depicts the atrialis side while the label “V” indicates the ventricularis side of the valve.

**Figure 4:**
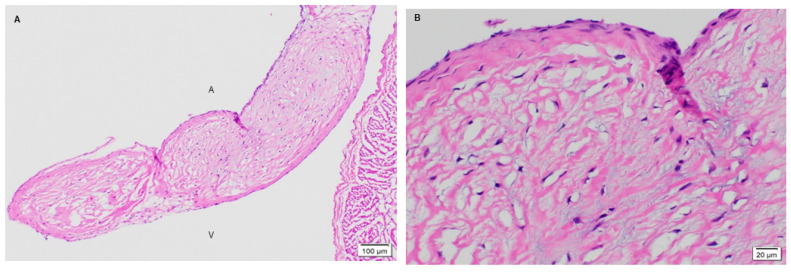
PSIS mitral valve explant at 3-month post-implantation in juvenile baboon stained with H&E. Mitral valve leaflet at lower magnification of 10× (**A**; H&E) with underlying myocardium. Higher magnification (**B**-40×) showing presence of interstitial cells and endothelium. In (**A**), the label, “A” depicts the atrialis side while the label “V” indicates the ventricularis side of the valve.

**Figure 5:**
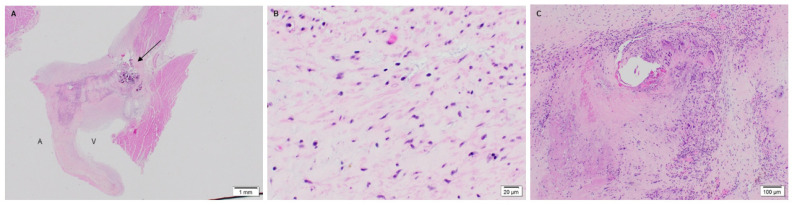
PSIS mitral valve explant at 11-month post implantation in juvenile baboon stained with H&E. Mitral leaflet and annulus at 1.25× (**A**–**C**) with calcification (**A**-arrow) and chronic inflammatory response including multinucleated giant cells (**C**-10×) but with cellular infiltration of neo-leaflet (**B**-40×) as well. In (**A**), the label, “A” depicts the atrialis side while the label “V” indicates the ventricularis side of the valve.

**Figure 6:**
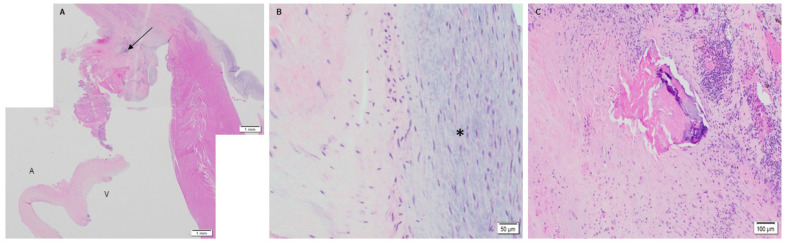
PSIS mitral valve explant at 20-month post implantation in juvenile baboon stained with H&E. Mitral valve leaflet and annulus at 1.25× (**A**–**C**) with neo-endocardial tissue growth (asterisk; **B**-20×) and calcification and chronic inflammation at the annulus (**C**-10×). In (**A**), the label, “A” depicts the atrialis side while the label “V” indicates the ventricularis side of the valve.

The native juvenile baboon mitral valve was used as the control, where H&E exhibited the native tissue morphology of the leaflet as well as its cellular content (Figure 3). The 3-month PSIS explanted mitral leaflet had a similar morphology to the native leaflet with some native cellular infiltration having occurred (Figure 4). Similarly, the 11-month PSIS valve explant had cellular infiltration occurring at the neoleaflet (Figure 5A,B). However, it was observed that there were areas on the leaflet that had residual PSIS that was unfilled with new tissues, resulting in a chronic inflammatory response at those sites (Figure 5C). Furthermore, calcification formed at the annulus of the valve (Figure 5A). The longest explant timepoint at 20 months demonstrated similar findings to the 11-month explant, wherein there were areas of physiological cellular infiltration at the neoleaflet (Figure 6A,B), but with concomitant chronic inflammatory responses at the sites of exposed and residual PSIS that remained unfilled with new tissues (Figure 6C). Finally, the annulus region of this explant also was found to have the presence of calcification at the annulus (Figure 6A).

#### 3.1.2. Phenotype of Native and Explanted PSIS Mitral Valve Leaflets

**Figure 7:**
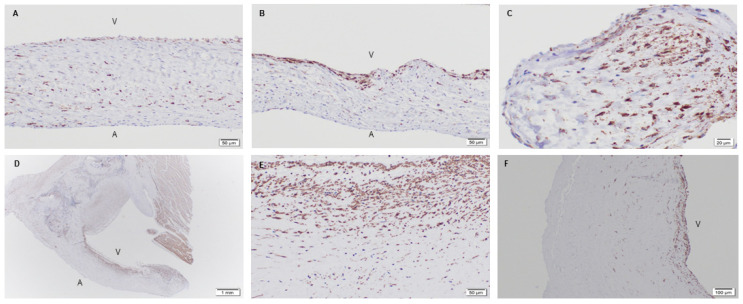
Native juvenile baboon mitral valve explant and PSIS mitral valve explants staining for α-SMA. Explants of the native mitral valve leaflets with the presence of α-SMA (smooth muscle cells) in brown (**A**,**B**; 20×) was observed throughout the leaflets but was predominant on the ventricularis (v) side. Presence of α-SMA (smooth muscle cells) in brown was also seen at the fragmented edge of the mitral valve leaflet (**C**-40×) explanted at 3-month post-implantation. The 11-month (**D**-1.25×, **E**-20×) and 20-month (**F**-10×) explants had the preferential presence of α-SMA (smooth muscle cells) stained in brown on the ventricular surface but with a distribution that occurred till its centerline location, similar to the native baboon leaflet. In (**A**–**D**,**F**), the label, “A” depicts the atrialis side while the label “V” indicates the ventricularis side of the valve.

**Figure 8:**
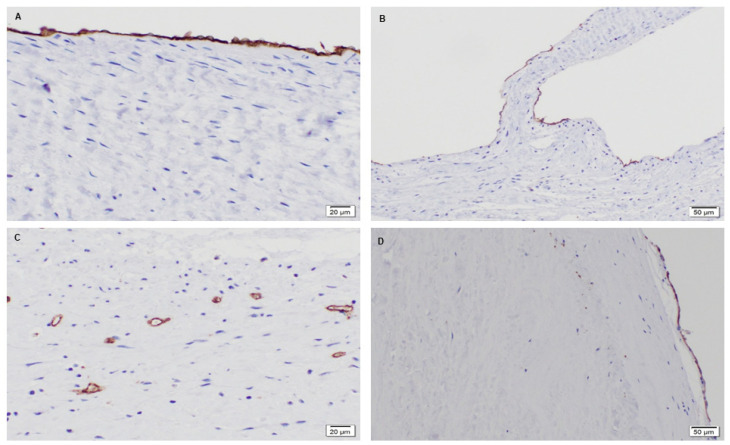
Native juvenile baboon mitral valve explant and PSIS mitral valve explants staining for CD31. The native leaflets had a presence of CD31 (endothelial cells) in brown that lined at the outer surface of the leaflet (**A**-40×, **B**-20×). For the 11-month explant, the presence of CD31 positive staining was present at the central region of leaflet (**C**-40×). Finally, the 20-month explant showed the presence of CD31 positive staining distribution at the surface of the leaflet (**D**-20×) similar to the native leaflet.

Given the formation of ECM and cellular infiltration, the valvular phenotype of recruited cells, such as interstitial (α-SMA) and endothelial (CD31) markers, were of importance. Immunohistochemistry for the valve phenotype were performed on all samples [40]. It was revealed that the native baboon mitral valve contained smooth muscle cells throughout the entire leaflet, mainly at the ventricularis layer (Figure 7A,B). Endothelial cells, on the other hand, were found to be present on the outer surface of the leaflets (Figure 8A,B). For the 3-month post-implantation explant, there was a presence of smooth muscle cells specifically at a fragmented edge of the explant (Figure 7C). The presence of endothelial cells (CD31) on the leaflet at this time point was not observed. In part, a sufficient number of endothelial cells may not have adhered or repopulated on the PSIS mitral valves at this early time point. In addition, it is possible that some cell loss may have occurred during valve explant handling and histological processing.

Interestingly, the later time points of 11 and 20 months post-implantation resembled the native baboon valve in terms of their smooth muscle cells distribution [40]. At 11 months post-implantation, the presence of smooth muscle cells was seen throughout the whole leaflet but preferentially on the ventricularis layer of the leaflet (Figure 7D,E), while the cells resided at the center of the leaflet in small vascular structures (Figure 8C). On the other hand, in the 20-month explant, both smooth muscle and endothelial cells were present preferentially on the outer surface of the valve (Figure 7F and Figure 8D), which resembled the native baboon mitral valve cellular distribution even more closely.

### 3.2. Extracellular Matrix Assessment via Spatial Intensity Quantifications on Explanted Mitral Valves

#### 3.2.1. Extracellular Matrix Assessment via Spatial Intensity Quantifications of Explanted Native and PSIS Mitral Valve Leaflet 

**Figure 9:**
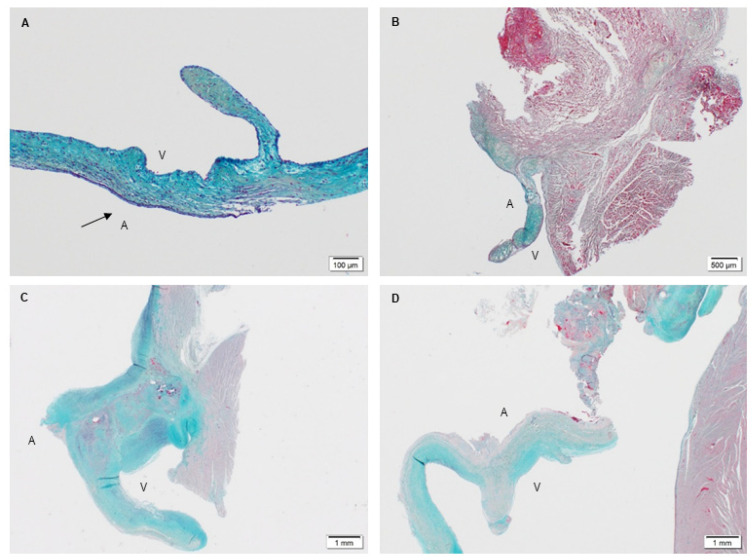
Movat’s histological stain to depict the ECM components of native juvenile baboon and PSIS mitral valve explants. The native mitral valve leaflet (**A**-10×) and the 3-month PSIS explant (**B**-2×) exhibited matrix consisting of collagen, elastin, and proteoglycans (yellow/green, black, and blue, respectively). Notable, there was evidence of elastin deposition along the atrialis layer (A-arrow). Both the 11- (**C**-1.25×) and 20-month (**D**-1.25×) PSIS explants exhibited similar matrix components (collagen, elastin and proteoglycans), but with the added presence of fibrin (dark pink). In (**A**–**D**), the label, “A” depicts the atrialis side while the label “V” indicates the ventricularis side of the valve.

**Figure 10:**
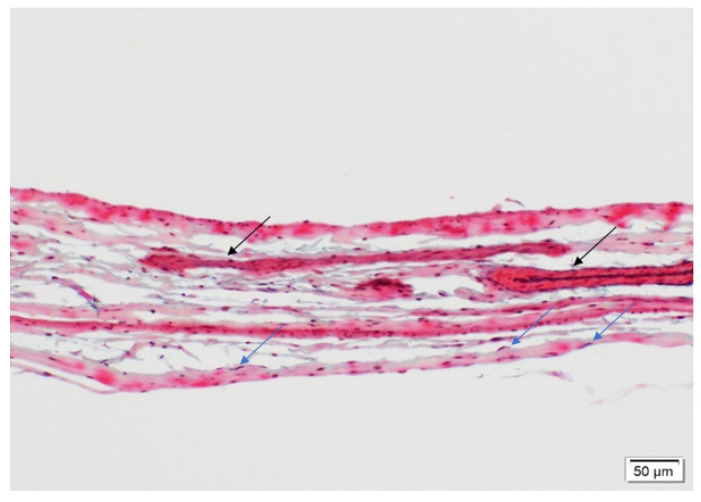
Movat’s histological stain showing the ECM components of a raw PSIS bioscaffold (Cormatrix, Roswell, GA, USA). The decellularized bioscaffold was predominantly composed of collagen (yellowish) with small vessels (black arrows) and scattered spindle cells (blue arrows; 20×). Spindle cells are fibroblasts that are long in shape and are a natural part of the body’s response to injury. These spindle cells are present before implantation.

**Figure 11:**
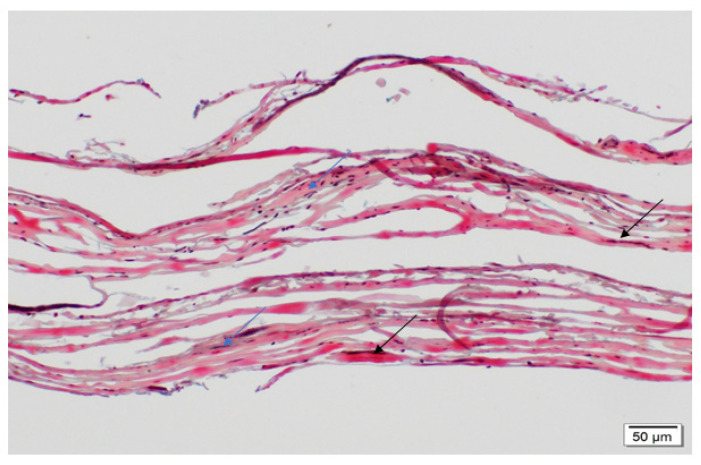
Movat’s histological stain showing the ECM components of an acellular PSIS bioscaffold (Cormatrix, Roswell, GA, USA). This version two, acellular bioscaffold, was predominantly composed of collagen (yellowish) with small vessels (black arrows) and scattered spindle cells (blue arrows; 20×). Spindle cells are fibroblasts that are long in shape and are a natural part of the body’s response to injury. These spindle cells are present before implantation. This version of PSIS has fewer cells than the decellularized version 1 seen in Figure 10, but the cells are still not completely removed.

Movat’s staining was performed on the explants along with the native baboon mitral valve (positive control) and the decellularized PSIS bioscaffold (negative control). Movat’s stains for collagen, elastin, proteoglycans, and fibrin correspond to green/yellow, purple/black, blue, and dark pink/red stains, respectively. The native mitral valve leaflets were composed of collagen, elastin and proteoglycans (Figure 9A). Interestingly, the elastin resided at both the atrialis and ventricularis sides of the leaflet, with collagen and proteoglycans that were distributed circumferentially throughout the entire leaflets (Figure 9A). The decellularized PSIS bioscaffold before implantation was mostly composed of collagen with muscle-like tissue and small vessels (Figure 10). The 3-month explant mimicked the native tissues of the baboon leaflet, i.e., trace amounts of elastin on the atrialis and ventricularis sides, with collagen and proteoglycans throughout the leaflets (Figure 9B) [40]. The 11- and 20-month explants mostly consisted of collagen and proteoglycans (Figure 9C,D) [40]. The 11-month explant was also found to be similar to the native and 3-month explants, wherein elastin was found on both sides of the leaflet (Figure 9C). However, calcified deposits and trace amounts of fibrin were also detected in the 11- and 20-month explants (Figure 9C,D). A second version of the decellularized PSIS, an “acellular” bioscaffold, was assessed as well demonstrating similar ECM structure as the previous version (Figure 10) but with a decrease of cellular content (Figure 11). Unfortunately, this version of the bioscaffold was not available until after our implantations had occurred.

**Figure 12:**
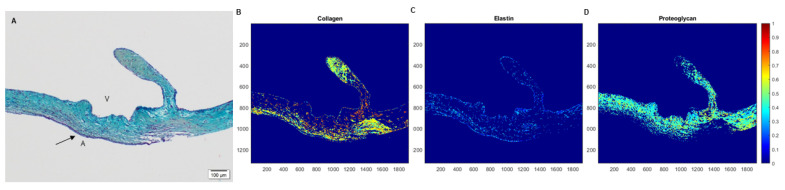
Spatial intensity maps to determine the ECM percent content. The movat’s histological stain of the native mitral valve (**A**-10×) is shown to demonstrate the spatial intensity maps computed for each image collected. Here, specifically, the matrix proteins of collagen (**B**), elastin (**C**), and proteoglycans (**D**) (yellow/green, black, and blue, respectively), were found to be 54%, 15%, and 31%, respectively, of each ECM protein. The collective data are shown in Table 1, Table 2 and Table 3 for the leaflets, annulus, and neochordae locations, respectively. In (**A**), the label, “A” depicts the atrialis side while the label “V” indicates the ventricularis side of the valve.

The spatial intensity mapping on Movat’s staining at the leaflet area (Figure 12) revealed no significant differences (*p* > 0.05) between the groups for any ECM proteins (collagen, *p* = 0.77; elastin, *p* = 0.28; proteoglycans, *p* = 0.26; fibrin, *p* = 0.05; Table 1) [40]. To determine the unfilled areas of the PSIS leaflet the average percent (%) of residual PSIS remaining was calculated. The unfilled residual PSIS was found to be in the order of 2% ± 0.01, 3% ± 0.12, and 4% ± 0.46 for 3-month, 11-month, and 20-month explants, respectively.

#### 3.2.2. Extracellular Matrix Assessment via Spatial Intensity Quantifications of Explanted PSIS Mitral Valve Annulus

**Figure 13:**
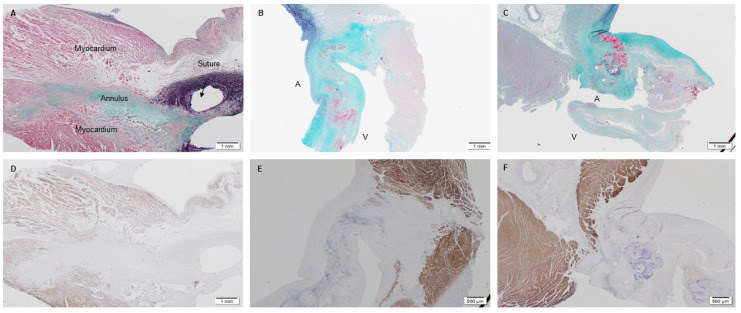
PSIS mitral valve explanted at 3-, 11-, and 20-month post-implantation focused on the annulus. The annulus region (**A**–**C**-1.25×; Movat’s) exhibited collagen, elastin, proteoglycans, and fibrin (yellow/green, black, blue, and dark pink, respectively). In (**B**,**C**), the label, “A” depicts the atrialis side while the label “V” indicates the ventricularis side of the valve. Tissue integration (**D**-1.25×, **E**,**F**-2×) was found to be evident between the annulus and the surrounding myocardium as shown via troponin staining in brown.

The next area of interest was the annulus area of the mitral valve. For this region, tissue formation and integration were assessed via Movat’s staining and immunohistochemistry of troponin, which stains for muscle tissue. As the native baboon annulus was not removed during the PSIS valve replacement surgical procedure, a positive control was not assessed. All the explanted PSIS valves showed tissue formation, with collagen, elastin, and proteoglycans present (Figure 13A–C) [40]. The 3-month explant was mostly composed of collagen at the annulus, while the 11- and 20-month explants showed comparable amounts of collagen and proteoglycans (Figure 13B,C). These later time point explants (11 and 20 months post-implantation), exhibited fibrin near the annulus (Figure 13B,C). Furthermore, the troponin stain confirmed seamless integration of the annulus to the myocardium for all the explants (Figure 13D–F).

Spatial intensity maps (Figure 13 and Table 2) indicated no significant difference (*p* > 0.05) between the groups for all ECM proteins at the annulus (collagen, *p* = 0.25; elastin, *p* = 0.10; fibrin, *p* = 0.55) except for proteoglycans (*p* = 0.02) [40]. In other words, the proteoglycans were found to be significantly lower (*p* < 0.05) in the 3-month explant compared to the 11- and 20-month explants (Table 2). The unfilled areas of PSIS at the annulus location were calculated to be 2% ± 0.79, 7% ± 0.10, and 3% ± 0.10 for 3-month, 11-month, and 20-month explants, respectively.

#### 3.2.3. Extracellular Matrix Assessment via Spatial Intensity Quantifications of Explanted PSIS Mitral Valve “Legs”

**Figure 14:**
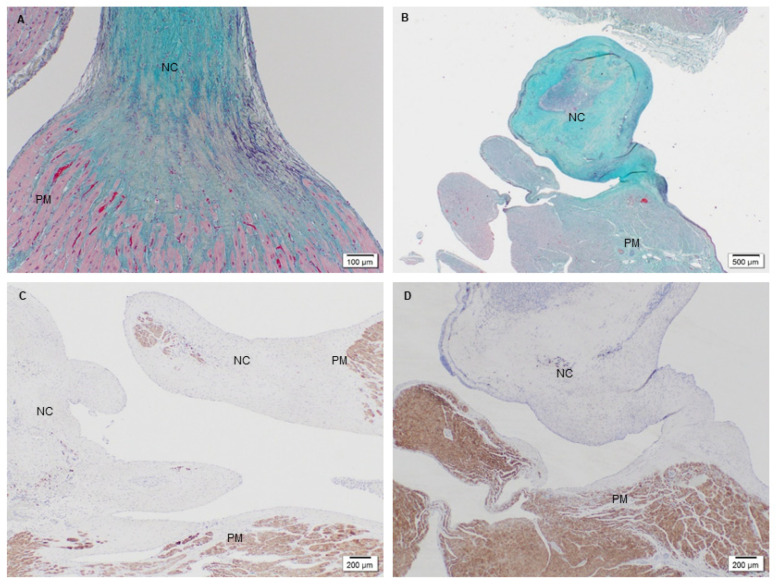
PSIS mitral valve explanted at 11- and 20-month post-implantation focused on the neochordae and papillary muscle point. The PSIS mitral valves exhibited neochordae formation (**A**–**C**-1.25×; Movat’s) consisting of collagen, elastin, proteoglycans, and fibrin (yellow/green, black, blue, and dark pink, respectively). Seamless tissue integration (**C**-1.25×, **D**-2×) between the neochordae and the surrounding papillary muscles (PM) as shown via troponin staining in brown, was achieved.

The last focal area of interest were the distal limbs or “legs” of the PSIS mitral valves (Figure 1). These legs consisting of PSIS were sutured onto the papillary muscles, thereby temporarily serving the function of chordae tendineae. These “legs” were found to be replaced by neochordae tissues. Tissue formation and integration in this region of the mitral valve apparatus were assessed similarly to the annulus location. However, it was not possible to assess the 3-month explant due to the limited tissue depth when histological sectioning at this early time point. Therefore, our assessments at this location focused solely on the 11- and 20-month explants of the PSIS neochordae. Both explants demonstrated all ECM proteins of collagen, proteoglycans and elastin (Figure 14A,B) and had seamlessly integrated into the papillary muscles (Figure 14C,D) [40]. The 11-month explant also showed trace amounts of fibrin.

Spatial intensity maps (Figure 14, Table 3) demonstrated that there was no significant difference (*p* > 0.05) between the groups for any ECM proteins (collagen, *p* = 0.41; elastin, *p* = 0.48; proteoglycans, *p* = 0.35; fibrin, *p* = 0.24) [40]. The unfilled areas of PSIS at the neochordae were calculated to be 1% ± 0.19 and 3% ± 0.04 for 11-month and 20-month explants, respectively.

## 4. Discussion

Annually, both acquired and congenital valvular heart diseases continue to increase globally [41]. For children specifically, this is a major concern due to an absence of small-sizing and a severe lack in support of somatic growth among current clinical treatments [41]. A tissue-engineered valve would therefore be a potentially promising approach especially for infants born with critical congenital heart valve diseases.

Decellularized tissues have recently gained renewed interest for cardiovascular applications since they contain the ECM and biological components, which may positively impact the cells and provide a platform for active tissue remodeling [41]. For the heart valve application, the viscoelastic mechanical properties of bioscaffolds are an added attraction in that they can facilitate normal valve dynamics in the acute term (3 months) [32]. In the present study, we focused on the de novo tissue that had formed following PSIS bioscaffold mitral valve replacement in a juvenile, non-human primate animal model via histological assessments and spatial intensity quantification. Specific amounts of various valve-relevant ECM components and cellular phenotypes on the PSIS mitral valve apparatus (annulus, leaflets and chordae tendineae) were evaluated at three valve explantation time points (3-, 11- and 20-month).

The PSIS mitral valves were able to regenerate the primary valve tissue ECM components of collagen, elastin and proteoglycans (Figure 9, Figure 13 and Figure 14). Furthermore, it was found that the PSIS mitral valve was recellularized with cells that exhibited the valve phenotype, specifically being positive for both smooth muscle cells (α-SMA) and endothelial cells (CD31) markers (Figure 7 and Figure 8). Progressive migration of the CD31 positive staining towards the PSIS valve surface appeared to have occurred with longer implant durations, with a similar surface distribution for CD 31 positive staining exhibited by the 20-month PSIS valve explant compared to the native baboon mitral valve leaflet (Figure 8). This similarity in positive CD31 staining solely at the leaflet surface is suggestive of PSIS valve endothelialization. However, the fact that this distribution was not observed in the 11-month PSIS explant does raise the concern of a very delayed formation of an endothelium. We interpret this to be a direct modulatory effect towards the chronic inflammatory responses that had initiated approximately 3 months following PSIS mitral valve implantation, noting that the presence of the CD31 marker is known to regulate inflammatory responses [42]. We had also previously reported that the PSIS mitral valves had functioned adequately up to 3 months as evidenced via echocardiography, with only trivial/mild regurgitation events during this timeframe [32]. Given that immune mediated responses are bounded by a complex balance that could either promote cardiovascular regeneration [43] or cause an adverse effect [44], we identified that in the case of the PSIS mitral valve, a conservative 3-month window exists in which additional processing steps (e.g., utilization of stem cell seeding) will be required to enable complete valve regeneration and integration in the host recipient within this timeframe. This 3-month window is a conservative estimate given the much faster rate of somatic growth in juvenile non-human primates compared to humans (5 years to adulthood in baboons as opposed to 18 years in humans). Additionally, given the evidence of uneven endothelialization in TEHVs [45], it would be important to confirm the distribution and phenotype of the cells on the TEHV leaflet surfaces via more detailed histological sectioning and additional staining (e.g., vWF), respectively. As a proof-of-concept study however, this verification was not conducted here.

At the leaflet location, all explants (3-, 11-, and 20-month) regenerated ECM proteins (Figure 9B–D) with no significant difference (*p* > 0.05) between the groups (Table 1) [40]; the PSIS leaflets were comparable in ECM composition to the native baboon mitral valve leaflets (Figure 9A). In addition to regeneration of valve tissues, later explantation time points (at 11 and 20 months) unfortunately also exhibited chronic inflammation and calcification (Figure 5 and Figure 6). This could be attributed to some porcine cells that were found to still be present on the PSIS bioscaffolds. Moreover, small areas (<7%) of the PSIS valve apparatus remained unfilled with de novo host tissues even after 20 months of implantation, despite having been decellularized, which may have increased their vulnerability to a hostile immune response (Figure 10). The concomitant presence of calcification and chronic inflammation using PSIS bioscaffold valves has also previously been reported in sheep and lambs that had the valves implanted in the pulmonary position for 2 months [39]. This suggests that calcification on bioscaffold valves is an intermediate rather than an acute event, which in our study occurred between 3 to 11 months post-mitral valve implantation and was likely associated with a hostile immune response. The initial hostile immune response (chronic inflammation) could have been triggered by the presence of some porcine cell residue that unknowingly had remained on the bioscaffold, following which, trace amounts of fibrin deposition were also observed (Figure 9, Figure 13 and Figure 14; Table 1, Table 2 and Table 3). This would be similar to a common wound healing response involving immune cells that migrate to the site of injury [46], except that in this case, the implanted PSIS mitral valve was the targeted destination. Nonetheless, the hostile immune response did not adversely affect the PSIS mitral valve function for the first 3 months following implantation. This suggests that a 3-month window is available to implement future processes with the use of bioscaffolds that could accelerate regeneration and permit complete TEHV integration in the host.

At the annulus of the PSIS mitral valve, regenerated ECM was found and had completely integrated with the myocardium (Figure 13) [40]. The lack of proteoglycans in the 3-month explant relative to the later explant time points (11 and 20 months; *p* < 0.05) could have in part led to the failure of the PSIS valve, which is of course biodegradable and thus necessitates an adequate rate of de novo tissue filling. Proteoglycans are not only important in contributing to valve’s mechanical properties, but, more importantly, play a central role in cell proliferation, differentiation, and development [47]. Like the PSIS leaflets, fibrin was present in the annulus region at the later explant time points (11 and 20 months; Figure 13B,C). Although fibrin deposition is commonly seen during wound healing, the presence of fibrin in the 20-month explant may correlate to endocarditis, that was the cause of its failure. Notably, endocarditis can lead to continuous inflammation due to lesions composed of fibrin [48]. It is important to point out here that the endocarditis appeared to be more of a hostile immune response to PSIS as a foreign material rather than to a foreign living organism, given the absence of cells with bacterial morphology in the H&E histology that was conducted on the PSIS valve explants (Figure 4 and Figure 6).

The neochordae of the PSIS mitral valve had fully regenerated, containing ECM proteins in all the PSIS valve explants (Figure 14A,B), with no significant difference (*p* > 0.05) between them (explanted after 3, 11, and 20 months, respectively; Table 3) [40]. Furthermore, the neochordae was found to have been recellularized and had completely and seamlessly integrated with the papillary muscles and the left heart wall (Figure 14C,D). This was possible simply with the use of raw PSIS bioscaffold material that served as the initial chordae tendineae structure (the “legs” component; Figure 1) at the time of implantation. This finding was found to be similar to complete re-endothelialization and recellularization events that have previously been shown to occur at attachments points [41]. Conversely, this may also explain why this was not observed for the valve leaflet surfaces, which are largely free structures [41].

Even though there were useful insights gained in this proof-of-concept study’s assessment of PSIS mitral valve’s somatic growth potential in a juvenile non-human primate model, there were nonetheless several limitations. Primarily, this was associated with a very small sample size of PSIS mitral valve explants (*n* = 1 baboon/time point), which prevented detailed statistical assessments of the histological and immunofluorescence outcomes. Moreover, another major limitation of the current study lies in the fact that specific immunological responses were not directly evaluated, which may be beneficial moving forward to determine how to mitigate the chronic inflammation occurring. Additional specific markers of interest for future studies would be for immune cells (e.g., CD45, CCR7, CD163, and T cells), confirmation of the endothelial phenotype (e.g., vWF) and additional evidence of calcification (e.g., von Kossa). Finally, the presence of a small amounts of porcine cell residue was found to be still present on the PSIS bioscaffolds that were utilized, which may have induced a hostile immune response. As such, the decreased presence of these porcine cells from the bioscaffold needs to be confirmed prior to any subsequent in vivo investigations, which we have now been able to confirm has improved with a smaller presence of these porcine cells (Figure 11) with more recent PSIS bioscaffold samples (Cormatrix Inc., Roswell, GA, USA).

In conclusion, PSIS bioscaffold mitral valves were successful in promoting host regeneration of ECM proteins and recellularization after implantation in growing, juvenile, non-human primate recipients. To develop a functional TEHV that mimics the native healthy heart valve, recapitulation of ECM proteins are important [49], which the PSIS bioscaffold mitral valves were able to induce, while supporting the somatic growth of the juvenile baboons. A major limitation of this approach, however, appears to be the chronic inflammation that occurs due to residual PSIS that remains unfilled with de novo valve tissues even though these regions are relatively a small portion of the entire valve implant (<7% in all cases). We believe that in vitro deposition of a thin layer of valve-relevant allogeneic ECM onto the raw bioscaffold valve prior to implantation (e.g., using stem cells), may help to overcome this issue by triggering ECM-induced native cellular chemotaxis and thus, accelerated and complete filling of de novo host valve tissues in the valve implant. This accelerated valve tissue regeneration will thereby substantially reduce the risk of an adverse host immune response and/or calcific deposition. Regardless of the specific approaches that are used for additional bioscaffold valve processing to accelerate tissue regeneration, we hereby conclude in this proof-of-concept study that 3 months is a safe estimate of the timeline in which complete regeneration of the valve and integration with the host needs to occur.

## Figures and Tables

**Table 1:** Average ECM components (%) of baboon native and PSIS mitral valve explanted at varying time points at the leaflet location. Approximately five images per time point were assessed to determine the average percentages of each ECM component. No significant differences (*p* > 0.05) were seen between any of the groups and in particular, comparing the PSIS explants to the native baboon valve.

Baboon Valve Type	Area	% Collagen	% Elastin	% Proteoglycans	% Fibrin
**Native Mitral Valve**	Leaflet	41% ± 0.07	12% ± 0.02	46% ± 0.07	-
**3-Month PSIS Explant**	Leaflet	45% ± 0.11	7% ± 0.02	24% ± 0.21	-
**11-Month PSIS Explant**	Leaflet	40% ± 0.07	11% ± 0.02	48% ± 0.06	1% ± 0.00
**20-Month PSIS Explant**	Leaflet	49% ± 0.06	9% ± 0.01	40% ± 0.04	3% ± 0.01

**Table 2:** Average ECM components (%) of PSIS mitral valve annulus explanted at varying time points. Approximately four images per time point were assessed to determine the average percentages of each ECM component. The ECM % contents varied at each explant time point with no apparent trend. *, ^+^ as indicated, signify a significant difference between the respective groups.

Baboon Valve Type	Area	% Collagen	% Elastin	% Proteoglycans	% Fibrin
**3-Month PSIS Explant**	Annulus	61%	12%	5% * ^+^	-
**11-Month PSIS Explant**	Annulus	41% ± 0.04	14% ± 0.02	44% ± 0.03 *	1% ± 0.00
**20-Month PSIS Explant**	Annulus	52% ± 0.07	6% ± 0.02	38% ± 0.05 ^+^	4% ± 0.03

**Table 3:** Average ECM components (%) of PSIS mitral valve neochordae and papillary muscles explanted at varying time points. Approximately seven images per time point were assessed to determine the average percentages of each ECM component. The ECM % contents varied at each explant time point with no apparent trend. No significant differences (*p* > 0.05) was seen between the 11- and 20-month PSIS mitral valve explants.

Baboon Valve Tye	Area	% Collagen	% Elastin	% Proteoglycans	% Fibrin
**11-Month PSIS Explant**	NC/PM	50% ± 0.06	11% ± 0.0	37% ± 0.07	2% ± 0.01
**20-Month PSIS Explant**	NC/PM	43% ±0.06	9% ± 0.01	47% ± 0.06	-

## Data Availability

The data presented in this study are available on request from the corresponding author.

## References

[1] Martin A.K., Weiner M.M., Feinman J.W., Bhatt H.V., Fritz A.V., Townsley M.M., Sharma A., Stawiarski K., Patel S.J., Zhou E.Y. (2021). The Year in Cardiothoracic and Vascular Anesthesia: Selected Highlights from 2020. J. Cardiothorac. Vasc. Anesth..

[2] Hinton R.B., Yutzey K.E. (2011). Heart valve structure and function in development and disease. Annu. Rev. Physiol..

[3] Henaine R., Roubertie F., Vergnat M., Ninet J. (2012). Valve replacement in children: A challenge for a whole life. Arch. Cardiovasc. Dis..

[4] Blitzer D., Herrmann J.L., Brown J.W. (2018). Pulmonary Autograft Mitral Valve Replacement (Ross II): Long-Term Follow-Up of a US Center. World J. Pediatr. Congenit. Hear. Surg..

[5] Soares A.L.F., van Geemen D., van den Bogaerdt A.J., Oomens C.W.J., Bouten C.V.C., Baaijens F.P.T. (2014). Mechanics of the pulmonary valve in the aortic position. J. Mech. Behav. Biomed. Mater..

[6] Cheung D.Y., Duan B., Butcher J.T. (2015). Bioprinting of Cardiac Tissues. Essentials of 3D Biofabrication and Translation.

[7] Sandusky G.E., Badylak S.F., Morff R.J., Johnson W.D., Lantz G. (1992). Histologic findings after in vivo placement of small intestine submucosal vascular grafts and saphenous vein grafts in the carotid artery in dogs. Am. J. Pathol..

[8] Robotin-Johnson M.C., Swanson P.E., Johnson D.C., Schuessler R.B., Cox J.L. (1998). An experimental model of small intestinal submucosa as a growing vascular graft. J. Thorac. Cardiovasc. Surg..

[9] Matheny R.G., Hutchison M.L., Dryden P.E., Hiles M.D., Shaar C.J. (2000). Porcine small intestine submucosa as a pulmonary valve leaflet substitute. J. Heart Valve Dis..

[10] Rosen M., Roselli E.E., Faber C., Ratliff N.B., Ponsky J.L., Smedira N.G. (2005). Small intestinal submucosa intracardiac patch: An experimental study. Surg. Innov..

[11] Ruiz C.E., Iemura M., Medie S., Varga P., Van Alstine W.G., Mack S., DeLigio A., Fearnot N., Beier U., Pavcnik D. (2005). Transcatheter placement of a low-profile biodegradable pulmonary valve made of small intestinal submucosa: A long-term study in a swine model. J. Thorac. Cardiovasc. Surg..

[12] White J.K., Agnihotri A.K., Titus J.S., Torchiana D.F. (2005). A stentless trileaflet valve from a sheet of decellularized porcine small intestinal submucosa. Ann. Thorac. Surg..

[13] Yavuz K., Geyik S., Pavcnik D., Uchida B.T., Corless C.L., Hartley D.E., Goktay A., Correa L.O., Timmermans H., Hodde J.P. (2006). Comparison of the endothelialization of small intestinal submucosa, dacron, and expanded polytetrafluoroethylene suspended in the thoracoabdominal aorta in sheep. J. Vasc. Interv. Radiol..

[14] Pavčnik D., Obermiller J., Uchida B.T., Van Alstine W., Edwards J.M., Landry G.J., Kaufman J.A., Keller F.S., Rösch J. (2009). Angiographic evaluation of carotid artery grafting with prefabricated small-diameter, small-intestinal submucosa grafts in sheep. Cardiovasc. Interv. Radiol..

[15] Bayrak A., Tyralla M., Ladhoff J., Schleicher M., Stock U.A., Volk H.-D., Seifert M. (2010). Human immune responses to porcine xenogeneic matrices and their extracellular matrix constituents in vitro. Biomaterials.

[16] Scholl F.G., Boucek M.M., Chan K.-C., Valdes-Cruz L., Perryman R. (2010). Preliminary experience with cardiac reconstruction using decellularized porcine extracellular matrix scaffold: Human applications in congenital heart disease. World J. Pediatr. Congenit. Hear. Surg..

[17] Gilbert C.L., Gnanapragasam J., Benhaggen R., Novick W.M. (2011). Novel use of extracellular matrix graft for creation of pulmonary valved conduit. World J. Pediatr. Congenit. Hear. Surg..

[18] Quarti A., Nardone S., Colaneri M., Santoro G., Pozzi M. (2011). Preliminary experience in the use of an extracellular matrix to repair congenital heart diseases. Interact. Cardiovasc. Thorac. Surg..

[19] Boni L., Chalajour F., Sasaki T., Snyder R.L., Boyd W.D., Riemer R.K., Reddy V.M. (2012). Reconstruction of pulmonary artery with porcine small intestinal submucosa in a lamb surgical model: Viability and growth potential. J. Thorac. Cardiovasc. Surg..

[20] Fallon A., Goodchild T., Gilbert C., Matheny R. (2012). A Pulmonary Valved Conduit of Porcine SIS Remodels into Native Tissue in an Ovine Model. Proceedings of the 5th Biennial Conference on Heart Valve Biology and Tissue Engineering.

[21] Poulin F., Horlick E., David T., Woo A., Thavendiranathan P. (2013). 3-Dimensional transesophageal echocardiography–guided closure of a gerbode shunt due to cormatrix patch dehiscence. J. Am. Coll. Cardiol..

[22] Yanagawa B., Rao V., Yau T.M., Cusimano R.J. (2014). Potential myocardial regeneration with CorMatrix ECM: A case report. J. Thorac. Cardiovasc. Surg..

[23] Zaidi A.H., Nathan M., Emani S., Baird C., del Nido P.J., Gauvreau K., Harris M., Sanders S.P., Padera R.F. (2014). Preliminary experience with porcine intestinal submucosa (CorMatrix) for valve reconstruction in congenital heart disease: Histologic evaluation of explanted valves. J. Thorac. Cardiovasc. Surg..

[24] Bibevski S., Scholl F.G. (2015). Feasibility and early effectiveness of a custom, hand-made systemic atrioventricular valve using porcine extracellular matrix (CorMatrix) in a 4-month-old infant. Ann. Thorac. Surg..

[25] Padalino M.A., Quarti A., Angeli E., Frigo A.C., Vida V.L., Pozzi M., Gargiulo G., Stellin G. (2015). Early and mid-term clinical experience with extracellular matrix scaffold for congenital cardiac and vascular reconstructive surgery: A multicentric Italian study. Interact. Cardiovasc. Thorac. Surg..

[26] Soucy K.G., Smith E.F., Monreal G., Rokosh G., Keller B.B., Yuan F., Matheny R.G., Fallon A.M., Lewis B.C., Sherwood L.C. (2015). Feasibility study of particulate extracellular matrix (P-ECM) and left ventricular assist device (HVAD) therapy in chronic ischemic heart failure bovine model. Asaio J..

[27] Mosala Nezhad Z., Poncelet A., De Kerchove L., Gianello P., Fervaille C., El Khoury G. (2016). Small intestinal submucosa extracellular matrix (CorMatrix^®^) in cardiovascular surgery: A systematic review. Interact. Cardiovasc. Thorac. Surg..

[28] Woo J.S., Fishbein M.C., Reemtsen B. (2016). Histologic examination of decellularized porcine intestinal submucosa extracellular matrix (CorMatrix) in pediatric congenital heart surgery. Cardiovasc. Pathol..

[29] Bibevski S., Levy A., Scholl F.G. (2017). Mitral valve replacement using a handmade construct in an infant. Interact. Cardiovasc. Thorac. Surg..

[30] Gonzalez B., Hernandez L., Bibevski S., Scholl F., Brehier V., Casares M., Bibevski J., Rivas K., Morales P., Wagner J. (2018). Recapitulation of human bio-scaffold mitral valve growth in the baboon model. Circulation.

[31] Gonzalez B., Perez M.G., Mirza A., Scholl F., Bibevski S., Wagner K.R., Bibevski J., Hernandez L.E., Ladich E., Brehier V. (2020). Extracellular Matrix Quantification of Fully Regenerated Neochorade After Bio-scaffold Mitral Valve Implantation in a Juvenile Non-human Primate Model. Circulation.

[32] Gonzalez B.A., Pour Issa E., Mankame O.V., Bustillos J., Cuellar A., Rodriguez A.J., Scholl F., Bibevski S., Hernandez L., Brehier V. (2020). Porcine small intestinal submucosa mitral valve material responses support acute somatic growth. Tissue Eng. Part A.

[33] Badylak S., Geddes L., Obermiller J. (2003). Extracellular matrix for myocardial repair. Hear. Surg. Forum.

[34] Slaughter M.S., Soucy K.G., Matheny R.G., Lewis B.C., Hennick M.F., Choi Y., Monreal G., Sobieski M.A., Giridharan G.A., Koenig S.C. (2014). Development of an extracellular matrix delivery system for effective intramyocardial injection in ischemic tissue. ASAIO J..

[35] Pavcnik D., Uchida B.T., Timmermans H.A., Corless C.L., O’Hara M., Toyota N., Moneta G.L., Keller F.S., Rösch J. (2002). Percutaneous bioprosthetic venous valve: A long-term study in sheep. J. Vasc. Surg..

[36] Witt R.G., Raff G., Van Gundy J., Rodgers-Ohlau M., Si M.-S. (2013). Short-term experience of porcine small intestinal submucosa patches in paediatric cardiovascular surgery. Eur. J. Cardio Thorac. Surg..

[37] Gerdisch M.W., Boyd W.D., Harlan J.L., Richardson J.B., Flack J.E., Palafox B.A., Johnson W.E., Sun B., Lee R., Guy T.S. (2014). Early experience treating tricuspid valve endocarditis with a novel extracellular matrix cylinder reconstruction. J. Thorac. Cardiovasc. Surg..

[38] Zafar F., Hinton R.B., Moore R.A., Baker R.S., Bryant R., Narmoneva D.A., Taylor M.D., Morales D.L. (2015). Physiological growth, remodeling potential, and preserved function of a novel bioprosthetic tricuspid valve: Tubular bioprosthesis made of small intestinal submucosa-derived extracellular matrix. J. Am. Coll. Cardiol..

[39] Van Rijswijk J.W., Talacua H., Mulder K., van Hout G.P., Bouten C.V., Gründeman P.F., Kluin J. (2020). Failure of decellularized porcine small intestinal submucosa as a heart valved conduit. J. Thorac. Cardiovasc. Surg..

[40] Gonzalez B.A. (2020). Bioscaffold Valve with and without Mechanically Conditioned Stem Cells for the Treatment of Critical Mitral Valve Diseases in the Young. Ph.D. Thesis.

[41] VeDepo M.C., Detamore M.S., Hopkins R.A., Converse G.L. (2017). Recellularization of decellularized heart valves: Progress toward the tissue-engineered heart valve. J. Tissue Eng..

[42] Vigne J., Bay S., Aid-Launais R., Pariscoat G., Rucher G., Sénémaud J., Truffier A., Anizan N., Even G., Ganneau C. (2019). Cleaved CD31 as a target for in vivo molecular imaging of inflammation. Sci. Rep..

[43] Hong H., Tian X.Y. (2020). The Role of Macrophages in Vascular Repair and Regeneration after Ischemic Injury. Int. J. Mol. Sci..

[44] Cutie S., Huang G.N. (2021). Vertebrate Cardiac Regeneration: Evolutionary and Developmental Perspectives. Cell Regener..

[45] Moreira R., Velz T., Alves N., Gesche V.N., Malischewski A., Schmitz-Rode T., Frese J., Jockenhoevel S., Mela P. (2015). Tissue-engineered heart valve with a tubular leaflet design for minimally invasive transcatheter implantation. Tissue Eng. Part C Methods.

[46] Ellis S., Lin E.J., Tartar D. (2018). Immunology of Wound Healing. Curr. Derm. Rep..

[47] Baasanjav S., Al-Gazali L., Hashiguchi T., Mizumoto S., Fischer B., Horn D., Seelow D., Ali B.R., Aziz S.A., Langer R. (2011). Faulty initiation of proteoglycan synthesis causes cardiac and joint defects. Am. J. Hum. Genet..

[48] Ashley E.A., Niebauer J. (2004). Cardiology Explained.

[49] Eslami M., Javadi G., Agdami N., Shokrgozar M.A., Eslami M., Javadi G., Agdami N., Shokrgozar M.A. (2015). Expression of COLLAGEN 1 and ELASTIN genes in mitral valvular interstitial cells within microfiber reinforced hydrogel. Cell J..

